# Innate Immunity to Respiratory Infection in Early Life

**DOI:** 10.3389/fimmu.2017.01570

**Published:** 2017-11-14

**Authors:** Laura Lambert, Fiona J. Culley

**Affiliations:** ^1^Faculty of Medicine, Respiratory Infections Section, National Heart and Lung Institute, Imperial College London, London, United Kingdom

**Keywords:** respiratory, neonatal, infection, respiratory syncytial virus, innate immunity

## Abstract

Early life is a period of particular susceptibility to respiratory infections and symptoms are frequently more severe in infants than in adults. The neonatal immune system is generally held to be deficient in most compartments; responses to innate stimuli are weak, antigen-presenting cells have poor immunostimulatory activity and adaptive lymphocyte responses are limited, leading to poor immune memory and ineffective vaccine responses. For mucosal surfaces such as the lung, which is continuously exposed to airborne antigen and to potential pathogenic invasion, the ability to discriminate between harmless and potentially dangerous antigens is essential, to prevent inflammation that could lead to loss of gaseous exchange and damage to the developing lung tissue. We have only recently begun to define the differences in respiratory immunity in early life and its environmental and developmental influences. The innate immune system may be of relatively greater importance than the adaptive immune system in the neonatal and infant period than later in life, as it does not require specific antigenic experience. A better understanding of what constitutes protective innate immunity in the respiratory tract in this age group and the factors that influence its development should allow us to predict why certain infants are vulnerable to severe respiratory infections, design treatments to accelerate the development of protective immunity, and design age specific adjuvants to better boost immunity to infection in the lung.

## Introduction

Respiratory infection is one of the leading causes of mortality in children under 5 years of age (1, 2). Early life respiratory viral infections are most commonly caused by rhinovirus, respiratory syncytial virus (RSV), influenza, parainfluenza virus, and coronavirus (3). Infection is frequently restricted to the upper respiratory tract but may develop into severe lower respiratory tract infection, such as RSV bronchiolitis, the leading cause of hospitalization of infants worldwide (4–7). Bacterial pneumonia in infants, caused by agents such as *Haemophilus influenzae* and *Streptococcus pneumoniae*, is estimated to cause a million deaths in infants under 5 years of age annually (8, 9). Maternal antibodies afford some protection against infection but wane over the first months of life, and neonates and infants respond poorly to vaccination, leaving early life as a window of particular vulnerability to respiratory infection (10, 11). Experiences during the crucial neonatal and infant window may shape respiratory health in the long term (12–14). Severe RSV infection in infants is associated with the development of wheeze and asthma in childhood (15–19) and even respiratory disease that occur late in life, such as chronic obstructive pulmonary disease, are associated with early life events (20–24).

At birth, the neonate emerges from the sheltered intrauterine environment into a plethora of antigenic challenges from pathogens, commensals, and harmless environmental antigens. Neonatal immunity is, in general, attenuated compared to that of adults (4, 25–29). Differences in immunity in early life are due to tissue leukopenia, cell intrinsic hyporesponsiveness, and inhibitory mechanisms, such as CD71+ immunosuppressive erythroid cells and high levels of adenosine in extracellular fluids (26, 28–31). Protective Th1 polarized responses and antibodies are produced less well in early life than in adults, along with a propensity to develop unwanted, Th2 or Th17 biased, or dysregulated inflammation (28, 31–33), for example, following vaccination or allergen exposure (34, 35). TLR stimulation of cord blood leukocytes results in a lower production of proinflammatory, Th1-associated cytokines (IL-12p70, TNF-α, IFN-α), and greater production of IL-10 and the Th17-promoting IL-6 and IL-23 when compared to stimulation of adult blood cells, although equivalent responses to TLR 7/8 ligand R848 occur (29, 36, 37). Over the first few years of life, antiviral and Th1-biasing cytokine production increases (38, 39).

**Figure 1:**
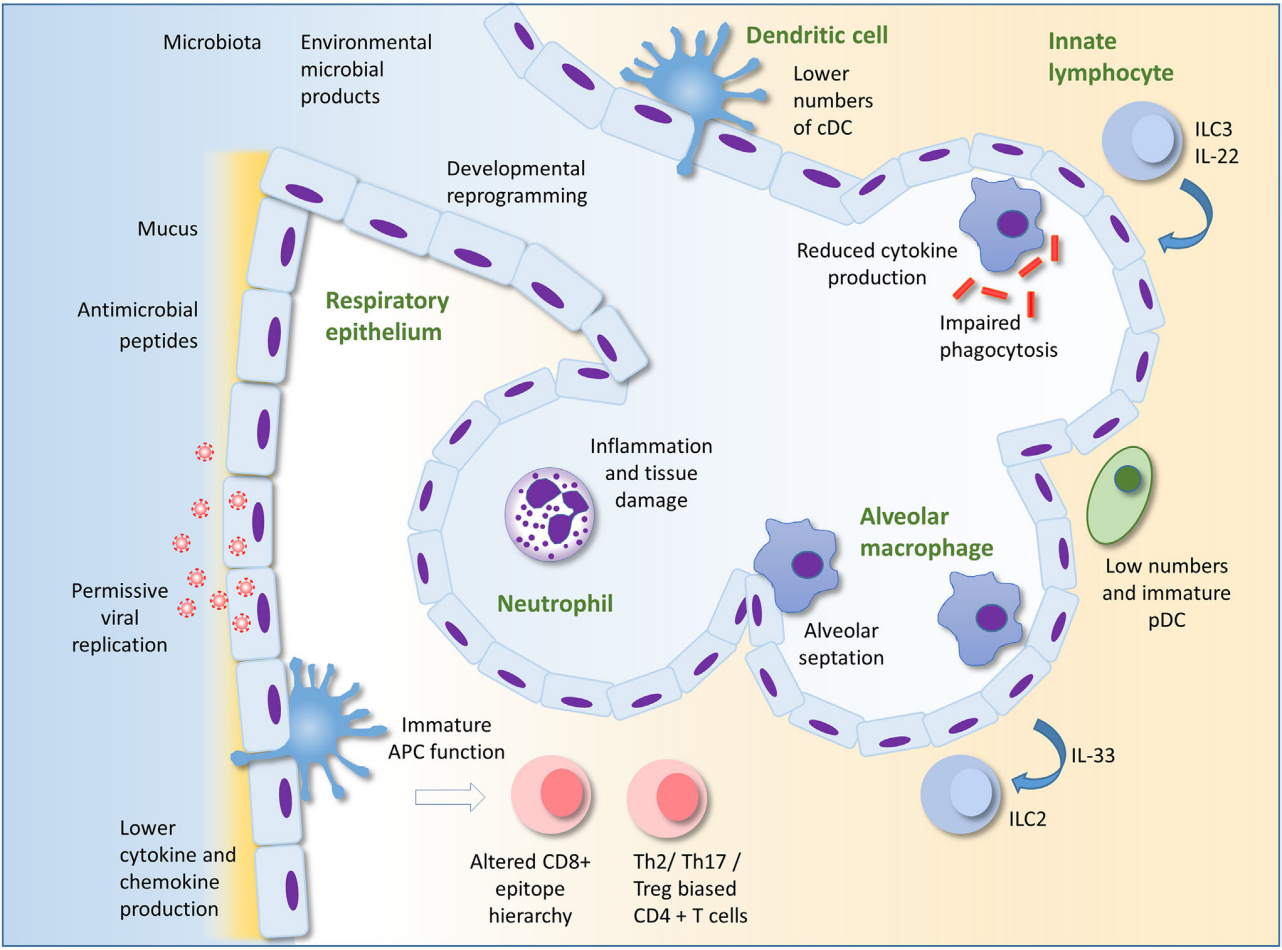
Innate immunity to infection in the lung in early life. Alveolar macrophages (AM) are the most numerous leukocyte in the lungs in early life. Reduced cytokine production and phagocytic ability in AM in early life compared to those of adults could underlie susceptibility to infection. AM also promote pre- and post-natal lung development and remodeling. The respiratory epithelium protects against infection through the production of mucus and antimicrobial peptides. Production of type I IFNs may be lower in infant than adult epithelial cells, perhaps permitting greater viral replication. Epithelial cells may interact with innate lymphocytes to both initiate and regulate inflammation. Developmental reprograming in the epithelium in early life may also alter the nature of the epithelial response to infection. There are low numbers of pDC in the lungs compared to adults. Recruitment of neutrophils to the lung occurs less readily in early life compared to adults in some circumstances, but in other situations, excessive recruitment of inflammatory cells can lead to lung inflammation, tissue damage, and impairment of gaseous exchange. Immaturity and lower numbers of dendritic cells, the environment as well as intrinsic differences in T cells in early life may result in the development of skewed helper T cell responses and an altered epitope hierarchy in CD8+ T cells. Innate immunity in the lung in early life is influenced by acquisition of the microbiota, exposure to microbial products and other environmental factors, as well as the infant genome. Adapted by permission from Macmillan Publishers Ltd: Nature Reviews Immunology (45), copyright 2014.

In the face of an inexperienced adaptive response, innate immunity is likely to play a more dominant role in protection against infection in early life than in adulthood. This is supported by the findings that many gene polymorphisms associated with severe RSV infection in infants encode components of the innate immune response (4, 40–43). The importance of TLR signaling in early life is illustrated by individuals with genetic deficiencies in components of the TLR signaling pathway such as MyD88 or IRAK-4. These patients are at high risk of bacterial infection in childhood, including in the respiratory tract; however, their condition improves dramatically with age (44). This review will focus on describing our current knowledge of innate immunity in the neonatal lung as a first line of defense against infection. Some potentially important mechanisms underlying susceptibility to lung infection in infants are summarized in Figure 1.

## Respiratory Immunity in Early Life

It is relatively difficult to obtain samples from the lower airways of healthy infant subjects, so many studies have been carried out in murine and other animal models. Information on the cellular composition of the neonatal lung in humans has come from analysis of bronchoalveolar lavage fluid composition (46–49), immunohistochemistry (50), and more recently, extensive phenotypic analysis of leukocyte subsets in pediatric tissues (51–53).

### Adaptive Immunity

Fetal airways are essentially devoid of lymphocytes, they are seeded from birth, and lymphocytes increase as a proportion of airway cells over the first few years of life (48, 54). There is a relative paucity in CD4+ cells (46, 50), and memory T cells are less abundant in infant lungs than in adults, though they are more abundant in the lungs than many other tissues (51). Tregs are relatively abundant in pediatric tissues and may have a higher suppressive capacity than those from adults (28, 51) and a transient increase in regulatory T cells, associated with microbial colonization, protects from hyperresponsiveness to allergen (35). A failure of regulation may underlie excessive inflammation in infection, as in RSV bronchiolitis (43), and RSV infection in early life can increase susceptibility to allergic inflammation in the mouse model through an impairment of regulatory T cells (4, 55). CD8+ T cells in the lung correlate with disease severity in infants with respiratory failure due to respiratory viral infection (52) and in neonatal mice infected with RSV, a CD8+ T cell epitope hierarchy emerges, which is distinct to that of adults (56). Distinct phenotypes of adaptive lymphocytes are found in early life. A subset of Th cells in human cord blood produce the neutrophil chemoattractant interleukin-8 upon activation (57) and, during RSV infection, a regulatory phenotype in the neonatal B cell compartment may dampen protective immunity (58).

### Lung Dendritic Cells (DCs)

There is some evidence that neonatal T cells have the capacity to mount adult-like protective responses to lung infection. Adoptive transfer of neonatal CD4+ T cells into *Pneumocystis carinii*-infected adult SCID mice allowed for adult-level pathogen clearance and cytokine production (59, 60), suggesting that the neonatal environment in the lung influences T cell responses. This may be due in part to the function of neonatal antigen-presenting cells. Neonatal mouse lungs contain relatively fewer conventional DCs (cDCs), which are immature and poorly functional (56, 61, 62), although mature functions *ex vivo* have been reported (63). During neonatal RSV infection, migratory cDCs are dominated by CD103+ DCs, while the CD11b+ contribution increases with age (64). These CD103+ DCs are phenotypically immature and poorly functional (65), and this may influence the magnitude and epitope hierarchy of the CD8+ T cell response (64–66), although these are also influenced by T cell intrinsic differences and regulatory T cells (56, 67). As well as stimulating protective responses, lung DCs in neonates must promote tolerance to harmless environmental antigens. CD11b+ cDCs in the lung induce Th2 responses to allergens, but transiently express high levels of PD-L1, which promotes tolerance, following acquisition of the microbiota (35, 68). In contrast to murine studies, the relative frequency of different DC subsets in the human lung appears to be relatively stable over the life course (53).

In the murine neonatal lung, potent IFN-α-producing pDC cells are scarce (61), and there is limited recruitment of pDCs and IFN-α production following RSV infection (69).

### Alveolar Macrophages (AM)

Lung resident macrophages, which include AM and the less well-characterized interstitial macrophages (70–72), are an important component of the first line of defense in the lung. In the steady state, AMs remove debris and maintain a tolerogenic environment; during infection, they secrete proinflammatory cytokines and contribute to pathogen clearance; and after infection, they aid resolution of inflammation (45). AMs are the predominant cell type in the neonatal airway, they appear in the alveolar compartment from just before birth and throughout the first week of life, and are relatively abundant and self-renewing, persisting for at least 11 weeks in mice (47–50, 73, 74).

Stimulation of cultured cells has been used to interrogate the relative antimicrobial functions of neonatal and adult AMs. LPS stimulation of rodent or ovine AMs results in similar or even enhanced upregulation of TNF-α and CXC-chemokines in neonatal compared to adult cells (75–77), though others demonstrated a reduced translocation of NF-κB to the nucleus of AM from neonatal mice (78). Enhanced phagocytosis by neonatal compared to adult rat AM has been observed (75), but others have reported impaired phagocytosis and subsequent killing of yeast particles in neonatal rhesus monkey AMs; and impaired phagocytosis of opsonized red blood cells in neonatal rat AMs in comparison to adults (79, 80). In a murine model of *Pneumocystis* infection, neonatal AMs were delayed in their expression of activation markers *in vivo* in comparison to adults (81). Similarly, during murine neonatal RSV infection, there was reduced and delayed AM activation compared to adult infection (82), but intranasal IFN-γ was able to promote AM maturation (82). Little is known about responses in human infant AMs. Cultured cells obtained by bronchoalveolar lavage from infants <2 years of age produce lower IL-1 and TNF-α following LPS stimulation compared with cells from children aged 2–17 (54). The apparent contradictions in the data on AM function in early life may reflect differences in the species, age, experimental conditions, and assays used. Various macrophage functions are likely to mature at different rates. Neonatal and adult AMs are likely to behave differently in their respective lung environments, which is a limitation of these *in vitro* studies.

### Respiratory Epithelial Cells

The respiratory epithelium is the principal site of replication of respiratory viruses. It is in close communication with AM and acts an immune sentinel producing inflammatory mediators, such as type I and III interferons, mucus, and antimicrobial proteins (45, 83). Relatively little is known about the immunological functions of the airway epithelium in early life. In cultured tracheobronchial epithelial cells from Rhesus macaques of different ages (infant, juvenile, and adult), IL-8 production on exposure to LPS positively correlated with age (84). Furthermore, epithelial cells from juveniles housed in filtered air produced higher cytokine responses than those in conventional housing suggesting the microbial richness of the environment may influence epithelial responsiveness. The same group demonstrated that infant Rhesus monkey primary epithelial cell cultures are more permissive for the H1N1 influenza virus than those from adult airways, while producing less IL-1α (85).

In humans, type I IFNs are detected at only low levels in the airways of RSV-bronchiolitic infants. This may be due to inhibition of the host anti-viral response by the viral non-structural proteins but alternatively may reflect the timing of sampling, and an IFN-induced gene signature is detectable in blood (86–88). Pediatric nasal and airway epithelial cells cultured from bronchial brushings are readily infected with RSV (89–91) and poor induction of type I IFNs by RSV is reflected in these cultures (92, 93). Instead, the type III interferon IL-29 (IFN-λ) is detected both in the airways of bronchiolitic infants and in cultures of RSV infected airway epithelial cells, and IL-29 pretreatment of cultured epithelial cells attenuates RSV growth (92, 93). Epithelial cells are probably a key source of inflammatory cytokines in respiratory tract secretions of infants with acute RSV (92, 94, 95), including the type-2 immunity promoting cytokine IL-33 (96). The cells used in many *in vitro* experiments on pediatric respiratory epithelial cells were originally taken from the conducting airway and data surrounding lower airway and ATII cells in early life is even sparser.

Antimicrobial proteins are a first line of defense at barrier sites and are produced primarily by epithelial cells and innate leukocytes, particularly neutrophils (97, 98). In the lung, they include surfactants as well as S100s, β-defensins, and cathelicidin and they may provide protection against important infant respiratory infections, including RSV (99–102). Cathelicidin has direct antiviral activity against RSV, can prevent infection *in vitro* and *in vivo* and in children hospitalized with bronchiolitis, those with low serum cathelicidin were significantly more likely to have RSV infection and a longer hospital stay (97, 103–107).

### Innate Lymphocytes

Neonatal murine lungs show no quantitative deficiency in γδ T cells as a proportion of CD3+ T cells (61, 108). Exposure to allergen in neonatal mice can stimulate innate ILC2 lymphocytes, a major source of type 2 cytokines (109). Colonization by the microbiota in neonates protects against the accumulation of potentially pro-inflammatory mucosal iNKT cells in the lung and gut (110). Colonization of the gut of neonatal mice can also lead to intestinal DC mediated upregulation of CCR4 on IL-22 producing ILC3, which allows their migration into the lungs of neonatal mice, and promotes protection against bacterial pneumonia (111).

### Neutrophils

Recruitment of innate leukocytes and, in particular, neutrophils, is likely to play an important role in the innate response to infection in the neonatal lung following microbial recognition. Both TLR4 gene and protein expression are present in the murine lung in the fetus and increase with age through to adulthood (112, 113). TLR2 expression is also present in the human fetal lung and increases with gestational age (114). It appears that there is an immaturity of chemokine production at baseline in the respiratory mucosa. Expression of CXCL2 is low in neonatal mice compared with adults (115) and in uninfected infants (newborn to 18 months), the concentration of IL-8 in nasal washes positively correlates with age (116). There is a dramatically reduced and delayed neutrophil influx in neonatal lung in response to administration of LPS or bacteria in comparison to adult animals (75, 117–119). In the neonatal murine lung, infection with the paramyxovirus Sendai virus results in a minimal early influx of neutrophils and low production of pro-inflammatory cytokines compared with the adult lung; similarly in murine RSV infection, early pro-inflammatory cytokine production is impaired (108, 115). Diminished recruitment of neutrophils may also be due to an impaired chemotaxic ability of infant neutrophils (25, 120, 121).

In severe RSV bronchiolitis in infants, neutrophils can account for the majority of cells recovered from the airways, associated with increased neutrophil elastase (122–125) and IL-8 (94, 126), although others have reported a lower inflammatory cytokine response in infants with severe vs mild RSV bronchiolitis (127). There is a considerable influx of neutrophils into *S. pneumoniae*-infected lungs of neonatal and adult mice, with the neonatal influx even occurring at a lower bacterial dose (128). It is unclear under what circumstances the neonatal lung will produce an equivalent or exacerbated inflammatory response compared to that of adults, whether this simply requires a high level of stimulation or whether additional factors are involved.

## Factors Influencing the Development and Maturation of Lung Immunity

Despite the apparent absence of a mature adult-like immune system, neonates are able to produce effective immune responses that defend against infection and indeed excessive inflammation can occur. The neonate must strike a balance between protection against infection and potential damage to the developing lung and may use alternative mechanisms of protection against infection to those that predominate in adults.

Exposure to microbial products from the environment, the microbiota, or infection may be beneficial in terms of their ability to promote immune maturation and more adult like innate and adaptive immunity (28, 30). Treatment with TLR agonists CpG or LPS during RSV infection alters the CD8+ T cell response toward a more adult-like immunodominance (66) and treatment of neonatal mice with CpG prior to RSV infection shifts the secondary response to re-infection away from a type 2 response (129). Furthermore, administration of BCG shifts lung CD4+ responses away from a Th2 bias and cDC from BCG treated lungs promote Th1 responses (61).

The microbiota is acquired from the mother at birth and in early life and an adult-like microbiome is established by around 3 years of age (130). The composition of the microbiota and microbial richness of the environment in which children develop have been linked to susceptibility to severe respiratory infections and the development of wheeze and asthma (131–133). Environmental microbial exposure may influence lung health by establishing the set-point of immunological responsiveness of the lung, as seen by the attenuation of allergic lung inflammation by airway exposure to LPS or endotoxin rich dust samples (133, 134). Additionally, commensal bacteria may influence neonatal respiratory immunity indirectly. For example, sensing of commensal bacteria by gut DCs promotes resistance to bacterial pneumonia in neonatal mice (111). Factors that shape the microbiota, such as delivery by cesarean section and antibiotic use in early life and pregnancy, are likely to profoundly influence the developing immune system (14, 135). Other environmental factors that regulate the balance of immunity in the infant respiratory tract may include diet, vitamin D status, breast feeding, maternal immunity, and exposure to environmental pollutants.

Significant stages of lung development occur both before and after birth and hyporesponsiveness to immune stimuli may have evolved to protect the developing lung from the disruptive and damaging effects of inflammation (136, 137). This is evidenced in mouse models of chorioamnionitis, where exposure of the fetal lung to LPS results in abnormal development of the distal airways (138, 139). In addition, IL-1β expression in the fetal or newborn lung impairs normal postnatal development (140). Reciprocally, the developmental programmes active in resident lung cells, which drive cell growth and differentiation may also influence immune responses (141, 142). Macrophages take on important roles in lung development and remodeling including septation and vascularization of the alveoli after birth (137, 143). Macrophages associate with sites of branching morphogenesis where they assume a tissue remodeling phenotype and promote development through production of growth factors and matrix metalloproteases (143). Polarization of macrophages away from this phenotype might, therefore, be a mechanism by which pro-inflammatory signals disrupt lung development (138, 140). As with lung macrophages, the respiratory epithelium will be subject to lung developmental programmes extending into the postnatal period, which regulate epithelial cell proliferation and differentiation, and these may potentially also alter epithelial immunological function. Foxa2 is an epithelially expressed member of the forkhead family of transcription factors. In the developing lung, it regulates epithelial differentiation and controls goblet cell hyperplasia. It also has immunoregulatory functions and limits type-2 immunity through inhibition of the cysteinyl LT signaling pathway (83, 141, 144).

## Conclusion

The mechanisms that regulate inflammatory responses to microbial stimulation in the lung need to be more fully elucidated. Increasing our knowledge of how the developing immune system responds to infectious challenge is of importance for development of neonatal vaccines and treatments for exaggerated respiratory inflammation during infection. In certain circumstances, the immune system in early life is capable of adult-level responses, and perhaps boosting responses in at-risk infants—in treatment for acute infectious disease or as adjuvant for vaccination—would be a beneficial protective strategy. Additionally, selectively harnessing the protective innate mechanisms that are already expressed at adult or greater than adult levels in the neonate could be a safe therapeutic method. Thus, while early life is clearly a period of immunological vulnerability for the developing lung, it is also an opportunity for effective intervention strategies, which could benefit respiratory health not only in infancy, but into adulthood.

## Author Contributions

LL researched the literature and wrote the review. FC wrote the review, edited, and updated it.

## Conflict of Interest Statement

The authors declare that the research was conducted in the absence of any commercial or financial relationships that could be construed as a potential conflict of interest.
